# Single retinal image for diabetic retinopathy screening: performance of a handheld device with embedded artificial intelligence

**DOI:** 10.1186/s40942-023-00477-6

**Published:** 2023-07-10

**Authors:** Fernando Marcondes Penha, Bruna Milene Priotto, Francini Hennig, Bernardo Przysiezny, Bruno Antunes Wiethorn, Julia Orsi, Isabelle Beatriz Freccia Nagel, Brenda Wiggers, Jose Augusto Stuchi, Diego Lencione, Paulo Victor de Souza Prado, Fernando Yamanaka, Fernando Lojudice, Fernando Korn Malerbi

**Affiliations:** 1 Fundacao Universidade Regional de Blumenau, Rua Antonio Veiga 140, Blumenau, 89030-903 SC Brazil; 2 Botelho Hospital da Visão, Rua 2 de Setembro, 2958, Blumenau, 89052-504 SC Brazil; 3 Phelcom Technologies, São Carlos, SP Brazil; 4 Bayer Healthcare – Brazil, São Paulo, SP Brazil; 5 Cell and Molecular Theraphy Center (NUCEL), School of Medicine, University of São Paulo, São Paulo, SP Brazil; 6 Department of Ophthalmology, Federal University of São Paulo (UNIFESP), São Paulo, SP Brazil

**Keywords:** Diabetes, Diabetic retinopathy, Handheld camera, Single image, Artificial intelligence

## Abstract

**Background:**

Diabetic retinopathy (DR) is a leading cause of blindness. Our objective was to evaluate the performance of an artificial intelligence (AI) system integrated into a handheld smartphone-based retinal camera for DR screening using a single retinal image per eye.

**Methods:**

Images were obtained from individuals with diabetes during a mass screening program for DR in Blumenau, Southern Brazil, conducted by trained operators. Automatic analysis was conducted using an AI system (EyerMaps™, Phelcom Technologies LLC, Boston, USA) with one macula-centered, 45-degree field of view retinal image per eye. The results were compared to the assessment by a retinal specialist, considered as the ground truth, using two images per eye. Patients with ungradable images were excluded from the analysis.

**Results:**

A total of 686 individuals (average age 59.2 ± 13.3 years, 56.7% women, diabetes duration 12.1 ± 9.4 years) were included in the analysis. The rates of insulin use, daily glycemic monitoring, and systemic hypertension treatment were 68.4%, 70.2%, and 70.2%, respectively. Although 97.3% of patients were aware of the risk of blindness associated with diabetes, more than half of them underwent their first retinal examination during the event. The majority (82.5%) relied exclusively on the public health system. Approximately 43.4% of individuals were either illiterate or had not completed elementary school. DR classification based on the ground truth was as follows: absent or nonproliferative mild DR 86.9%, more than mild (mtm) DR 13.1%. The AI system achieved sensitivity, specificity, positive predictive value, and negative predictive value percentages (95% CI) for mtmDR as follows: 93.6% (87.8–97.2), 71.7% (67.8–75.4), 42.7% (39.3–46.2), and 98.0% (96.2–98.9), respectively. The area under the ROC curve was 86.4%.

**Conclusion:**

The portable retinal camera combined with AI demonstrated high sensitivity for DR screening using only one image per eye, offering a simpler protocol compared to the traditional approach of two images per eye. Simplifying the DR screening process could enhance adherence rates and overall program coverage.

**Supplementary Information:**

The online version contains supplementary material available at 10.1186/s40942-023-00477-6.

## Background

The screening of diabetic retinopathy (DR) is a milestone for the prevention of blindness and is recommended by many countries as well as the World Health Organization [1]. Successful screening strategies worldwide are usually based on color fundus photographs (CFPs), such as the English program [1]. However, blindness secondary to diabetes is still an unmet need in most low- and middle-income countries [2] and also in some high-income countries: in the USA, rates of screening as low as have been reported [3].

Solutions for increasing screening rates include public health policies, health education [2] and technological breakthroughs which may render the process simpler and more cost-effective. In that sense, the incorporation of telemedicine protocols, handheld devices, and artificial intelligence (AI) have all shown to increase the efficiency of screening [4]. Recently, autonomous AI systems have been granted regulatory approval for the detection of DR based on the analysis of two retinal images per eye [5, 6].

The imaging protocol for DR screening has gone through an evolution over the last decades, from the original ETDRS protocol of 7 fields until the widely accepted protocol of two retinal images per eye [7]. Simpler protocols have been associated with increased adherence, ultimately contributing to a program´s efficiency [7]. The challenge is to balance a simpler protocol without losing image quality and diagnostic accuracy. A protocol based on a single image per eye may save significant examination time in high-burden settings, such as mass screening campaigns, where more than one thousand people are screened for DR in a single morning. Such protocol may also be suitable for a staged mydriasis strategy: due to pupillary reflex secondary to the camera flash, the second image is harder to obtain without pharmacological mydriasis. In that sense, the ungradable rate is expected to be higher with two photos.

Our objective was to evaluate the performance of a DR screening protocol that employed a single retinal photo per eye, obtained with a handheld retinal camera and evaluated by an embedded AI system.

## Methods

### Study design, population and setting

This retrospective study enrolled a convenience sample of individuals aged over 18 years old with a previous type 1 or type 2 diabetes mellitus (DM) diagnosis who were summoned to attend the Blumenau Diabetes Campaign, a DR screening strategy that occurred from February to November 2021 at the city of Blumenau, Southern Brazil. The study protocol was approved by the ethics Committee of Fundação Universidade Regional de Blumenau (#39352320.5.0000.5370) and was conducted in compliance with the Declaration of Helsinki, following the institutional ethics committees. After signing informed consent, participants answered a questionnaire for demographic and self-reported clinical characteristics: age, gender, income, profession, educational level, type of diabetes, and diabetes duration. After answering the questionnaire, patients underwent ocular imaging.

### Imaging acquisition and grading

Imaging acquisition protocol and expert reading are detailed elsewhere [8]. Briefly, smartphone-based hand-held devices (Eyer, Phelcom Technologies LLC, Boston, MA) were used for the acquisition of two images of the posterior segment—one centered on macula and another disc centered (45° field of view)—for each eye, after mydriasis induced by 1% tropicamide eye-drops. Image acquisition was performed by a team of previously trained medical students, at public primary care health units. Human image reading was performed in a store-and-forward fashion at EyerCloud platform (Phelcom Technologies LLC, Boston, MA) by a single retinal specialist (FMP) after anonymization and quality evaluation. This ground truth analysis by a human grader was performed using two images per eye. Classification of DR was given per individual, considering the most affected eye, according to the International Council of Ophthalmology Diabetic Retinopathy (ICDR) classification. Patients with ungradable fundus images had their anterior segment evaluated for cataracts or other media opacities. No information other than ocular images was available for the reader, and the human grader was masked to the automated evaluation described below.

### Automated detection of DR

Images corresponding to one macula-centered image of each eye, were graded by an AI system trained with the Kaggle Diabetic Retinopathy dataset (EyePACS) and transfer learning with a dataset of approximately 16,000 fundus images captured using Phelcom Eyer. The system was previously validated for the detection of more than mild DR (mtmDR), details of which have already been described by our group elsewhere [8]. Only individuals who had images with enough quality were included in the analysis. In brief, the system is a modified version of the convolutional neural network (CNN) Xception, the input having been changed to receive images of size 699 × 699 × 3 RGB, with two fully connected layers of 2100 neurons added at the top: two neurons with softmax activation classifying the network input according to class. Softmax normalized the respective neuron input values, creating a probabilistic distribution in which the sum will be 1; the prediction corresponding to the interval between 0 and 1, indicating the likelihood of DR.

**Fig. 1:**
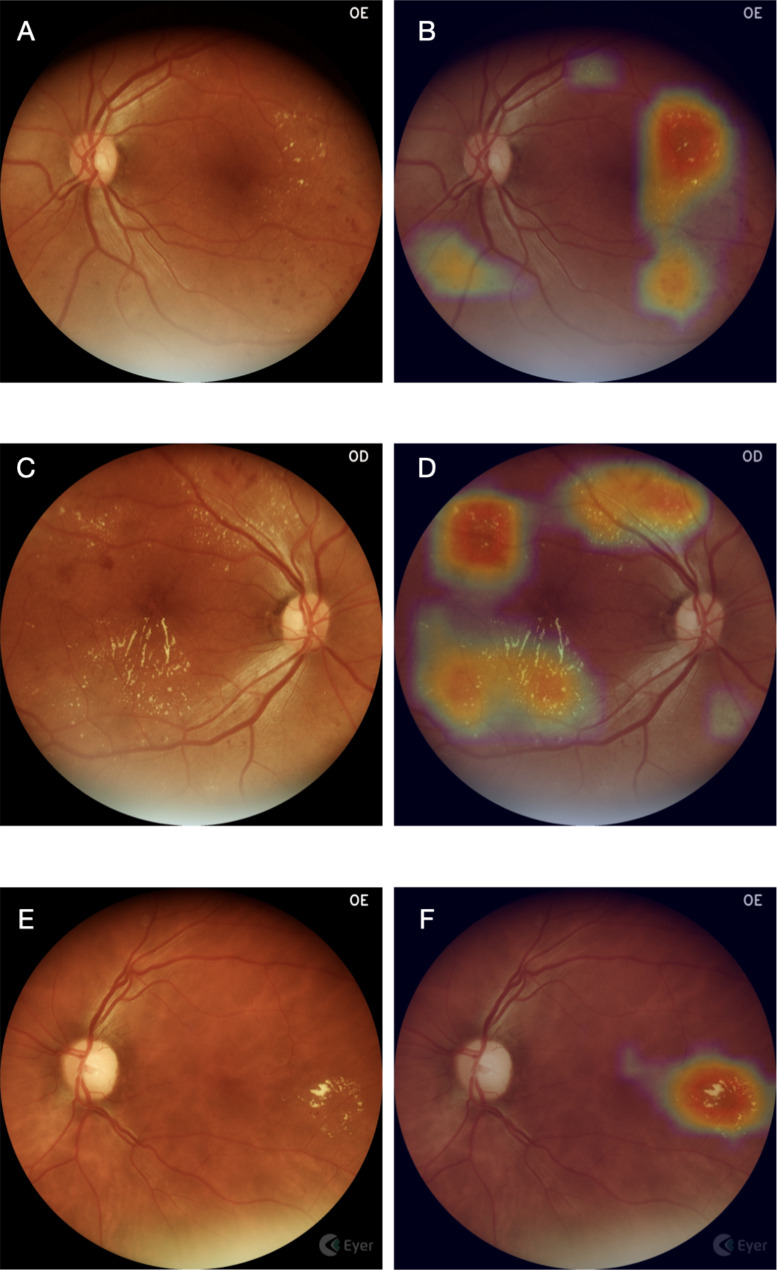
Example of heatmap visualization. (A, C and E) Color fundus photograph depicting clinical signs of diabetic retinopathy such as hard exudates and hemorrhages. (B, D and F) Overlay with the heatmap visualization can help identify lesions, flagged in a color scale, from blue (low importance) to red (high importance)

To visualize the location of the most important regions obtained by CNN, to discriminate between classes, the Gradient-Based Class Activation Map (GradCam) was used; it generates a heat map (EyerMaps, Phelcom Technologies LLC, Boston, MA) which highlights the detected changes (Fig. 1).

### Statistical analysis

Data were collected in MS Excel 2010 files (Microsoft Corporation, Redmond, WA, USA). Statistical analyses were performed using SPSS 19.0 for Windows (SPSS Inc., Chicago, IL, USA). Individual’s characteristics and quantitative variables are presented in terms of mean and standard deviation (SD). Sensitivity, specificity, positive predictive value (PPV), and negative predictive value (NPV), and their 95% confidence intervals (CIs), were calculated for the device outputs with no or mild DR and mtmDR compared with the corresponding reference standard classifications; comparison was made against human reading as the ground-truth; expert reading was based on the analysis of two retinal images per eye, while AI output considered only a single, macula-centered image per eye. The 0.3 threshold was chosen as the operating point (see Supplementary Material). Diagnostic accuracy is reported according to the Standards for Reporting of Diagnostic Accuracy Studies (STARD) [9].

## Results

Digital fundus photography images were obtained for both eyes of 817 individuals, 131 of whom (16%) could not be automatically analyzed due to insufficient quality. The remaining 686 individuals (average age 59.2 ± 13.3 years old, 56.7% women) met the inclusion criteria and had their images analyzed by the automatic system. Diabetes duration was 12.1 ± 9.4 years. Rates of insulin use, daily glycemic monitoring and treatment for systemic hypertension were 68.4%, 70.2% and 70.2%, respectively. Even though 97.3% of patients knew about the risk of blindness due to diabetes, 52.3% of patients underwent their first retinal examination during the event. The majority (82.5%) relied exclusively on the public health system. Individuals who were illiterate or who had not completed elementary school were 43.4%. DR classification according to the ground truth was as follows (Table 1): absent 68.1%, Nonproliferative (NP) Mild DR 19.1%, NP Moderate DR 6.8%, NP Severe DR 2.0%, Proliferative DR 4.5%; diabetic macular edema was detected in 7.7%.

**Table 1:** Distribution Among Patients of Diabetic Retinopathy Classification and Diabetic Macular Edema33

Diabetic Retinopathy Classification	%
Absent	68.1
NP mild DR	19.1
NP moderate DR	6.8
NP severe DR	2.0
Proliferative DR	4.5
Diabetic Macular Edema	7.7

NP: non proliferative; DR: diabetic retinopathy

### Artificial intelligence system performance

**Fig. 2:**
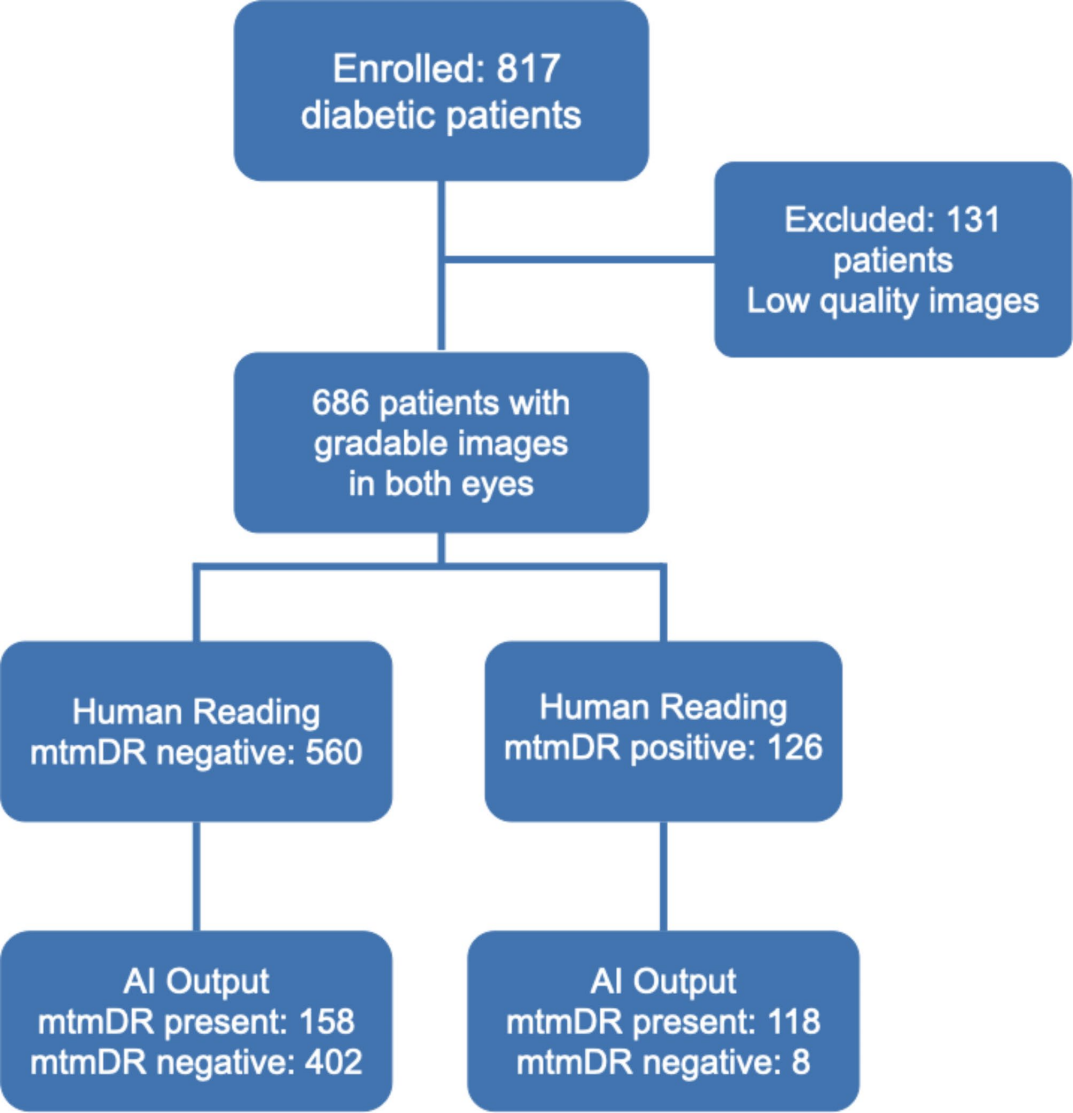
Standards for Reporting of Diagnostic Accuracy Studies (STARD) diagram for the algorithm mtmDR output. PPV and NPV for mtmDR were 65.4% (95% CI 62.2–68.5) and 95.2% (95% CI 91.0-97.5), respectively

The sensitivity/specificity, per the human grading standard, for the device to detect mtmDR was 93.6% (95% CI 87.9–97.2)/71.8% (95% CI 67.9–75.4), the Confusion Matrix is presented in Table 2. Figure 2 depicts the Standards for Reporting of Diagnostic Accuracy Studies (STARD) diagram for the algorithm mtmDR output. PPV and NPV for mtmDR were 42.7% (39.3–46.2), and 98.0% (96.2–98.9), respectively. Area under the receiver operating characteristic (ROC) curve was 0.86.

**Table 2:** Confusion Matrix for Reference Standard According to Human Grading and Device Output

	Human Grading mtmDR Positive	Human Grading mtmDR Negative
AI Output mtmDR Positive	118	158
AI Output mtmDR Negative	8	402

Legend: mtmDR = more than mild diabetic retinopathy

## Discussion

We herein report the results of automatic analysis for the detection of mtmDR with a single retinal image, obtained with a portable smartphone-based retinal camera. A high sensitivity (sensitivity 91,27%) had already been previously described for algorithmic evaluation of a two-image protocol with the same device [8]. The high sensitivity of the embedded AI system in our real-world sample compares well with previous reports of other automated systems [5, 10, 11]. The first two AI systems approved by the FDA for DR screening rely on protocols of two retinal images per eye and use traditional, tabletop retinal cameras: Idx DR [5] and EyeArt [6], and a recent study that validated seven AI systems for DR screening in the real world based on protocols of two retinal images per eye found sensitivities ranging from 50.98 to 85.90%, and specificity from 60.42 to 83.69% [11].

Portable handheld, low-cost retinal cameras have the potential to broaden the reach of DR screening programs, widening geographic areas and reaching populations that otherwise would not be screened by traditional methods [12]; such aspects potentially increase program´s efficiency due to higher coverage and increased adherence. A handheld device with integrated AI analysis has been reported in a screening performance with four fundus images per eye [13] with sensitivity of 95.8% and specificity of 80.2% for the detection of any DR. Another recent study with a handheld device and the same AI system, but a protocol of five fundus images per eye, reported a sensitivity of 87.0% and specificity of 78.6% for the detection of referable DR [14].

We have studied the performance of AI on a protocol based on a single fundus image per eye. It has been established that, regarding expert human reading, a single image protocol loses diagnostic accuracy in comparison to a two-images protocol [15]. However, with automatic reading, performance was considered satisfactory for screening, with the obvious advantages of obtaining one single image per eye; efforts to facilitate the process and make it less time-consuming are warranted to increase efficiency. Interestingly, macula-centered images have been considered to correspond to the most important region for deep learning systems in the evaluation of DR [16]. We have attained comparable diagnostic accuracy in comparison with the results reported by Nunez do Rio and colleagues [17]: their performance of a Deep Learning algorithm for the detection of referable DR analyzing only one retinal image per eye was as follows: sensitivity of 72.08% and specificity of 85.65%, corresponding to our threshold 0.85 (see Supplementary Material 1). The performance of our strategy also compares well with results from trained human readers when analyzing one image per eye [18].

Comparing to other algorithms, it has a relatively low specificity 71.8% (95% CI 67.9–75.4). However, it’s important to note that this is not an autonomous system, and retina specialists review the images before referring patients for an in-person evaluation. This approach helps to minimize costs while improving the specificity of the method through evaluation by specialists for those patients who truly require it. In this same strategy, Xie demonstrated that assistive and non-autonomous systems exhibit greater cost-effectiveness when compared to purely autonomous systems [19]. Improving the algorithm technology may increase this specificity without losing its main characteristic of a high sensitivity method for mass screening programs.

Another important aspect to discuss is the pupil status for retinal imaging, this might affect the number of ungradable images and AI performance. Piyaseana and cols reported that the proportion of ungradable images in non-mydriatic settings was 18.6% compared to 6.2% in mydriatic settings [20]. The present study was conducted in a mass screening program and pupil dilation was performed to ensure a faster imaging acquisition. It’s true that pupil dilation reduces the number of ungradable images and may increase algorithm performance. One strategy could be to use the staged mydriasis and dilate just those patients where image quality was not sufficient without pupil dilation.

The PPV reported in the present study was 42.7%, and the NPV was 98.1%; for the purpose of screening, high NPV is important to ensure that negative cases indeed do not have DR, while a low threshold for unclear cases possibly leads to low PPV [11]. A recent study that validated seven AI systems for DR screening in the real world based on protocols of four retinal images per eye found PPVs ranging from 36.46 to 50.80% and NPV from 82.72 to 93.69% [11]; a heterogeneous distribution of PPVs among different datasets has been attributed to differences in disease prevalence, on the basis of Bayes theorem [11]: sites with higher prevalence rates had higher PPV; of note, our sample had the majority of patients with no signs of disease (68%), which may account for the relatively low PPV.

Regarding the population evaluated in the present study, even though the screening was performed on a State that presents the 3rd highest human development index (HDI) of the country [21], over half of participants had their first fundus evaluation during this initiative, despite having a diabetes duration of 12.1 ± 9.4 years, evidencing that access to healthcare also lacks in such high-ranked settings of a middle-income country. Brazil is considered to host the sixth biggest population of individuals with diabetes worldwide [22]; being a country with continental dimensions and heterogeneous realities, Brazil also has many differences regarding social and economic aspects. As an example, a comparison between data collected in Blumenau (Southern Brazil) and Itabuna, situated in Bahia state (Northeastern Brazil), ranked 22nd for HDI, shows significant differences on the health profile of patients who underwent DR screening: the present sample from Blumenau, consisting of 686 individuals aged 59.2 ± 13.3 years, with average diabetes duration of 12.1 years, reported use of insulin in 68.4%; more than mild DR was present in 12.8%; and educational level was up to elementary school in 43.3%. In contrast, a sample of 940 individuals with diabetes from Itabuna aged 60.8 + 11.4 years, with average diabetes duration of 10.4 years, reported use of insulin in 25.8%; more than mild DR was present in 25.7%; and educational level was up to elementary school in 54.4% [15].

Despite the southern region of Brazil being one of the most developed in the country, with the municipality of Blumenau boasting one of the highest Human Development Index (HDI) levels nationwide, access to early detection of diabetic retinopathy remains highly limited. The need for mass screening programs highlights the population’s lack of access to DR evaluation. Thus, implementing mass screening programs and potentially incorporating regular and continuous assessment utilizing portable cameras in primary healthcare facilities could help decrease waiting times and improve access. This approach would serve as an effective strategy to mitigate diabetes-related blindness cases. A sentence has been included in the discussion to address this aspect.

We believe the main strength of this study is to present an automatic system with a potential to yield a high sensitivity for DR screening after evaluation of a single retinal image per eye; of note, the sensitivity attained was higher than the pre-specified endpoint for FDA approval of an automatic DR screening system [5]. Further steps for a DR screening program that would deploy the present tool could include acquisition of a second fundus image per eye only for detected cases, thereby rendering the screening process simpler for most patients, who would only need one image; further studies are needed to investigate this hypothesis.

Our study has several limitations, the most notable of which is that human grading was performed by only one specialist, a potential source of bias. Additionally, automatic evaluation was performed only on images with sufficient quality, limiting partially our conclusions regarding the real world, when a considerable rate of patients has ungradable images, mainly due to cataracts. Furthermore, diabetic maculopathy was not evaluated with gold standard methods; instead, its presence was inferred in non-stereoscopic images. Finally, the lack of comprehensive clinical and laboratory data is also a limitation of the current study.

This study presents a new concept of a single-image approach for diabetic retinopathy screening. However, due to its methodological limitations, particularly the fact that it had only one evaluator, its results need to be interpreted with caution. A high sensitivity prototocol was obtained for DR screening with a portable retinal camera and automatic analysis of only one image per eye. Further studies are needed to clarify whether a simpler strategy as compared to the traditional, two images per eye protocol, could contribute to superior patient outcomes, including increased adherence rates and increased overall efficacy of DR screening programs.

## Electronic supplementary material

Below is the link to the electronic supplementary material.

Supplementary Material 1

## Data Availability

Not applicable.

## Acronyms

AI: Artificial intelligence
CFPs: Color fundus photographs
CNN: Convolutional neural network
CIs: Confidence intervals
DM: Diabetes melittus
DR: Diabetic retinopathy
GradCam: Gradient-Based Class Activation Map
ICDR: International Council of Ophthalmology Diabetic Retinopathy
mtm: More than mild
NPV: Negative predictive value
NP: Non-proliferative
PPV: Positive predictive value
SD: Standard deviation
