# Supplementary material for: Single retinal image for diabetic retinopathy screening: performance of a handheld device with embedded artificial intelligence
Source: Int J Retina Vitreous. 2023 Jul 10;9:41. doi: 10.1186/s40942-023-00477-6 (PMC10332010; doi:10.1186/s40942-023-00477-6)
Supplement: Supplementary file 1 — Supplementary Material 1 [file 40942_2023_477_MOESM1_ESM.docx]

Supplementary Material

| **ROC** | **Sensitivity** | **Specificity** |
| --- | --- | --- |
| 0,00 | 100% | 0% |
| 0,05 | 98,00% | 55,88% |
| 0,10 | 94,67% | 63,38% |
| 0,15 | 94,00% | 67,65% |
| 0,20 | 93,33% | 69,41% |
| 0,25 | 92,00% | 71,91% |
| 0,30 | 90,67% | 73,82% |
| 0,35 | 89,33% | 75,29% |
| 0,40 | 88,67% | 76,47% |
| 0,45 | 88,00% | 77,65% |
| 0,50 | 85,33% | 79,56% |
| 0,55 | 84,00% | 80,74% |
| 0,60 | 83,33% | 81,76% |
| 0,65 | 79,33% | 82,35% |
| 0,70 | 77,33% | 83,24% |
| 0,75 | 76,00% | 84,12% |
| 0,80 | 74,67% | 84,85% |
| 0,85 | 72,00% | 85,59% |
| 0,90 | 70,67% | 86,76% |
| 0,95 | 66,00% | 87,50% |
| 1,00 | 0,00% | 100,00% |
